# Tracheostomy procedures in the intensive care unit: an international survey

**DOI:** 10.1186/s13054-015-1013-7

**Published:** 2015-08-13

**Authors:** Maria Vargas, Yuda Sutherasan, Massimo Antonelli, Iole Brunetti, Antonio Corcione, John G. Laffey, Christian Putensen, Giuseppe Servillo, Paolo Pelosi

**Affiliations:** Department of Neurosciences, Odonthostomatological and Reproductive Sciences, University of Naples, “Federico II”, Naples, Italy; Division of Pulmonary and Critical Care Medicine, Faculty of Medicine Ramathibodi Hospital, Mahidol University, 270 RAMA VI road, Bangkok, 10400 Thailand; Department of Intensive Care and Anaesthesiology, Università Cattolica del Sacro Cuore, Rome, Italy; Department of Surgical Sciences and Integrated Diagnostics, IRCCS San Martino IST, University of Genoa, Largo Rosanna Benzi 8, Genoa, 16131 Italy; AORN Ospedale dei Colli, Naples, Italy; Department of Anesthesia, Critical Illness and Injury Research Centre, Keenan Research Centre for Biomedical Science, St Michael’s Hospital, University of Toronto, Toronto, Canada; University of Bonn, Bonn, Germany

## Abstract

**Introduction:**

Percutaneous dilatational tracheostomy (PDT) is one of the most frequent procedures performed in the intensive care unit (ICU). PDT may add potential benefit to clinical management of critically ill patients. Despite this, no clinical guidelines are available. We sought to characterize current practice in this international survey.

**Methods:**

An international survey, endorsed and peer reviewed by European Society of Intensive Care Medicine (ESICM), was carried out from May to October 2013. The questionnaire was accessible from the ESICM website in the ‘survey of the month’ section.

**Results:**

429 physicians from 59 countries responded to this survey. Single step dilatational tracheostomy was the most used PDT in ICU. Almost 75 % of PDT’s were performed by intensive care physicians. The main indication for PDT was prolonged mechanical ventilation. Tracheostomies were most frequently performed between 7–15 days after ICU admission. Volume control mechanical ventilation, and a combination of sedation, analgesia, neuromuscular blocking agents and fiberoptic bronchoscopy were used. Surgical tracheostomy was mainly performed in ICU by ENT specialists, and was generally chosen when for patients at increased risk for difficult PDT insertion. Bleeding controlled by compression and stoma infection/inflammation were the most common intra-procedural and late complications, respectively. Informed consent for PDT was obtained in only 60 % of cases.

**Conclusions:**

This first international picture of current practices in regard to tracheostomy insertion demonstrates considerable geographic variation in practice, suggesting a need for greater standardization of approaches to tracheostomy insertion.

**Electronic supplementary material:**

The online version of this article (doi:10.1186/s13054-015-1013-7) contains supplementary material, which is available to authorized users.

## Introduction

Percutaneous dilatational tracheostomy (PDT) is one of the most frequent procedures performed in the intensive care unit (ICU) [1]. PDT may add potential benefit to clinical management of critically ill patients by increasing patients’ comfort, reducing the need for sedation, facilitating the weaning process and hastening ICU discharge [2]. Despite this, no clinical guidelines have been developed to delineate the best practice for this invasive and potentially risky procedure [3]. Recently the use of a customized checklist, adapted from the World Health Organization surgical safety checklist, has been suggested to improve the safety of PDT [4]. However, the utility of this checklist has not been demonstrated. To date, no study has investigated the tracheostomy practice at a multinational level. This is the first international survey aiming to evaluate the daily practice of PDT according to different settings, operators, types, timing, indications, procedural features, sedation and ventilation protocols, and intra-procedural, early and late complications. This survey may show a proof of concept that an international daily shared clinical practice on PDT techniques is possible.

## Materials and methods

The European Critical Care Research Network (ECCRN) of the European Society of Intensive Care Medicine (ESICM) endorsed this survey. Ethical approval for this study was not requested because this was a voluntary survey and no individual patient data were collected.

The questionnaire (Additional file 1) included 31 questions organized in two main sections, analyzing: 1) the profile of respondents and 2) the settings, operators, type, timing, indication, procedural features, sedation and ventilation protocol, and intra-procedural, early and late complications of PDT. The questionnaire was validated by a local panel of experts in intensive care and then underwent the peer review process by the ESICM Research Committee. After the acceptance by the ESICM Research Committee, the survey was carried out from May to October 2013, and was accessible from the ESICM website in the ‘survey of the month’ section. All ESICM members were alerted by email and invited to complete the survey via the ESICM research updates. All the data collected from the questionnaires were analyzed anonymously.

Submitted questionnaires were excluded from analysis if the respondents completed section II only. Failure to answer to one or more questions in section II was not a reason for exclusion.

The data were evaluated as total distribution of answers and then divided according to the geographical area of respondents within Europe (E) and outside Europe (OE) by using descriptive statistics. Data have been reported as counts and percentages. The effects of geographical location on PDT practices were analyzed using the chi-squared (*X*^2^) test for categorical variables or univariate analysis of variance (ANOVA) for continuous variables. Data have been weighted for the total number of participating intensive care units in and outside Europe, to perform tests of statistical significance. The *p* value was set at 0.05. Statistical analysis was performed using SPSS, version 20.0.

## Results

### Study respondents

Responses were received from 429 physicians from 59 countries (Europe 73.6 %, Asia 15.8 %, America 9.1 %, Africa 1.0 %, Australia 0.5 %) (Additional file 2). According to our entry criteria, 281 satisfactorily completed questionnaires from 52 countries (Europe 75.0 %, Asia 16.4 %, America 7.5 %, Africa 0.7 %, Australia 0.4 %) were included in the final analysis. Of the satisfactorily completed questionnaires, 75 % (n = 208) came from E while 25 % (n = 73) were from OE.

Table 1 shows the baseline characteristics of study respondents. The main specialty area was intensive care in 71.9 % of respondents (n = 202) followed by anesthesiology (22.4 %, n = 61). Public hospitals (45.6 %, n = 128) and university hospitals (42.7 %, n = 120) were the institutions most involved in the responses from E but not from OE (E vs OE: *X*^2^ = 52.573, *p* = 0.000). The type of ICU involved in responses was mixed ICUs (73.3 %, n = 206) followed by surgical ICUs (11 %, n = 31) from E but not OE, where medical ICUs (17.8 %, n = 13) followed mixed ICUs (71.2 %, n = 52) (E vs OE: *X*^2^ = 23.108, *p* = 0.000). The majority of respondents worked in an ICU with 6–10 beds (35.9 %, n = 101) in E but not OE, where the majority of responses came from ICU with more than 21 beds (28.8 %, n = 21) (E vs OE: *X*^2^=14.942, *p* = 0.011). The most frequent number of patients admitted to study ICUs annually was between 301 and 600 (34.9 %, n = 98) in E and OE (E 34.1 %, OE 37 %, E vs OE: *X*^2^ = 2.517, *p* = 0.472). Physicians in E performed two tracheostomy techniques more frequently, while in OE they performed one technique more frequently (E 52.4 %, n = 109; OE 49.3 %, n = 36; *X*^2^ = 19.708, *p* = 0.001).

**Table 1:** Baseline characteristics of study respondents

	Response rate			Statistics
	Total	Europe	Outside Europe	
Valid respondents	281 (100 %)	208/281 (74 %)	73/281 (26 %)	-
Main specialty area				
- Intensive care	202/281 (71.9 %)	147/208 (70.7 %)	55/73 (75.3 %)	
- Anesthesiology	63/281 (22.4 %)	51/208 (24.5 %)	12/73 (16.4 %)	
- Pulmonology	5/281 (1.8 %)	3/208 (1.4 %)	2/73 (2.7 %)	*X* ^2^ = 10.810
- Cardiology	2/281 (0.7 %)	0	2/73 (2.7 %)	*P* = 0.094
- Neurology	1/281 (0.4 %)	1/208 (0.5 %)	0	
- Surgery	2/281 (0.7 %)	1/208 (0.5 %)	1/73 (1.4 %)	
- Other	6/281 (2.1 %)	3/208 (1.4 %)	1/73 (1.4 %)	
Type of institution				
- Public hospital	128/281 (45.6 %)	105/208 (50.5 %)	23/73 (31.5 %)	*X* ^2^ = 52.573
- Private hospital	33/281 (11.7 %)	9/208 (4.3 %)	24/73 (32.9 %)	*P* = 0.000
- University hospital	120/281 (42.7 %)	94/208 (45.2 %)	26/73 (35.6 %)	
Type of intensive care				
- Cardiac	9/281 (3.2 %)	8/208 (3.8 %)	1/73 (3.8 %)	
- Mixed	206/281 (73.3 %)	154/208 (74 %)	52/73 (71.2 %)	*X* ^2^ = 23.108
- Medical	22/281 (7.8 %)	9/208 (4.3 %)	13/73 (17.8 %)	*P* = 0.000
- Neurological	13/281 (4.6 %)	12/208 (5.8 %)	1/73 (1.4 %)	
- Surgical	31/281 (11 %)	25/208 (12 %)	6/73 (8.2 %)	
Number of ICU beds				
- ≤ 5	8/281 (2.8 %)	7/208 (3.4 %)	1/73 (1.4 %)	
- 6−10	101/281 (35.9 %)	84/208 (40.4 %)	17/73 (23.3 %)	*X* ^2^ = 14.942
- 11−15	61/281 (21.7 %)	44/208 (21.2 %)	17/73 (23.3 %)	*P* = 0.011
- 16−20	49/281 (17.4 %)	32/208 (15.4 %)	17/73 (23.3 %)	
- ≥21	62/281 (22.1 %)	41/208 (19.7 %)	21/73 (28.8 %)	
Number of patients/years admitted in ICU				
- ≤300	39/281 (13.9 %)	31/208 (14.9 %)	8/73 (11 %)	*X* ^2^ = 2.517
- 301−600	98/281 (34.9 %)	71/208 (34.1 %)	27/73 (37 %)	*P* = 0.472
- 601−999	63/281 (22.4 %)	44/208 (21.2 %)	19/73 (26 %)	
- ≥1000	81/281 (28.8 %)	62/208 (29.8 %)	19/7 (26 %)	
Different techniques performed				
- one	99/281 (35.2 %)	63/208 (30.3 %)	36/73 (49.3 %)	
- two	14/281 (49.8 %)	109/208 (52.4 %)	31/73 (42.5 %)	*X* ^2^ = 19.708
- three	31/281 (11 %)	26/208 (12.5 %)	5/73 (6.8 %)	*P* = 0.001
- four	7/281 (2.5 %)	7/208 (3.4 %)	0	
- five	2/281 (0.7 %)	2/208 (1 %)	0	
- six	2/281 (0.7 %)	1/208 (0.5 %)	1/73 (1.4 %)	

### Tracheostomy

**Fig. 1:**
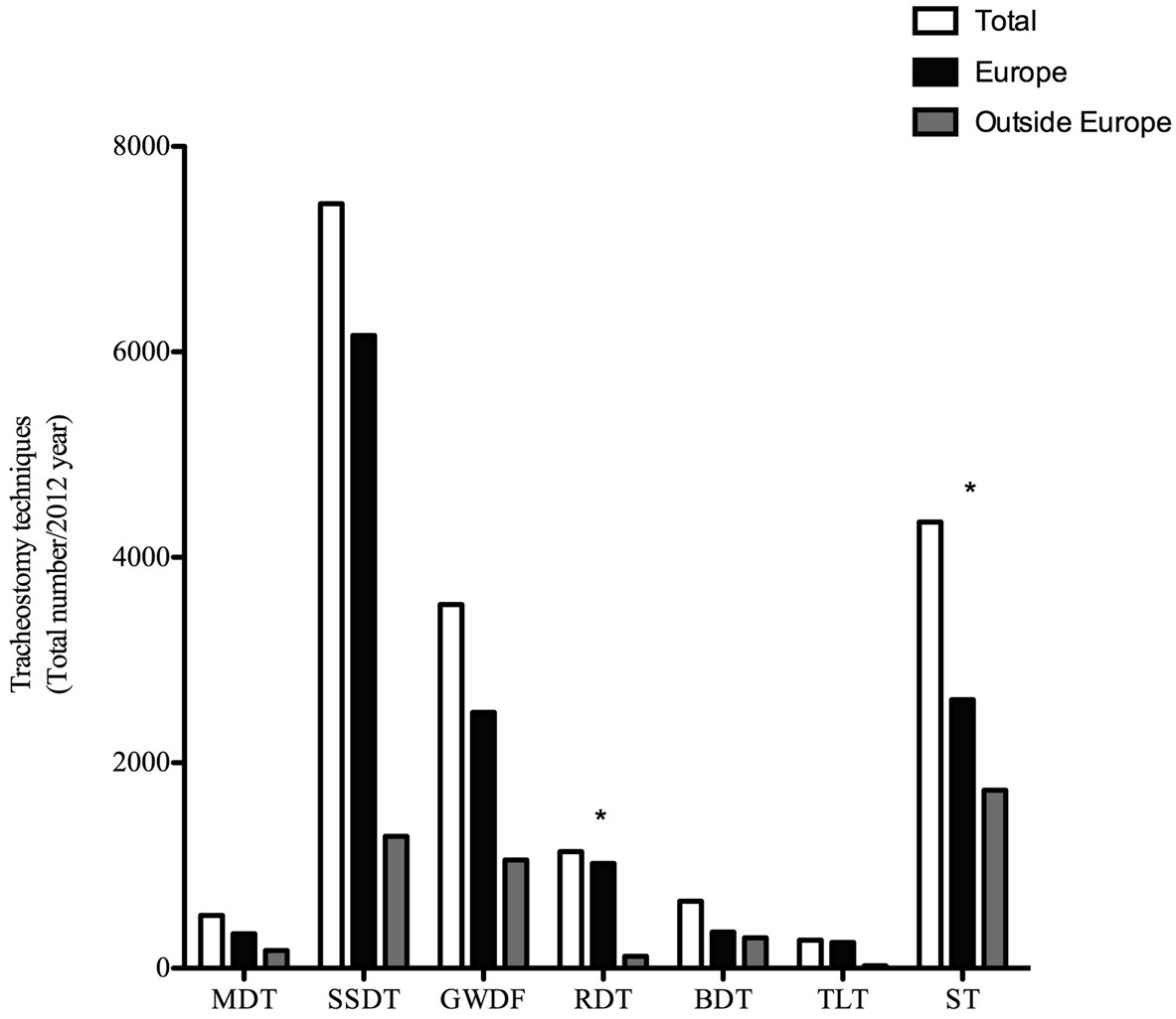
Distribution of different tracheostomies shown as the total number performed in and outside Europe. *Statistically significant. *MDT* multiple dilation tracheostomy, *SSDT* single-step dilation tracheostomy, *GWDF* guidewire dilating forceps, *RDT* rotational dilation tracheostomy, *BDT* balloon dilation tracheostomy, *TLT* translaryngeal tracheostomy, *ST* surgical tracheostomy

The total number of tracheostomies performed by the respondents was 17,894, with 74 % (n = 13,220) in E and 26 % (n = 4,764) in OE. Figure 1 shows the distribution of different tracheostomies. The most frequently performed tracheostomy procedure was the single-step dilation tracheostomy (SSDT) (41.6 %, n = 7,442) followed by surgical tracheostomy (ST) (24.1 %, n = 4,345). The most frequently performed tracheostomy procedure in E was the SSDT (46.6 %, n = 6,160) while OE it was the ST (36.4 %, n = 1,733).

### Informed consent

Informed patient/substitute decision-maker consent for tracheostomy was obtained in 61.2 % (n = 172) of participating ICUs. The informed consent was obtained more frequently OE than in E (informed consent obtained: OE 87.7 %, n = 64; E 51.9 %, n = 108; informed consent not obtained: OE 12.3 %, n = 9; E 48.1 %, n = 100; *X*^2^ = 49.332, *p* = 0.000).

### Percutaneous dilatational tracheostomy (PDT)

Table 2 describes the variations in approach to the performance of PDT in ICUs across the world. PDT in the ICU was more often performed by (one or more) intensivists (74 %, n = 208). The most frequent indication for PDT was prolonged mechanical ventilation (53.7, 151) followed by difficult or prolonged weaning 24.2 %, n = 68). The most frequent timing of PDT was between 7 and 15 days (54.4 %, n = 153). The majority of respondents used volume control ventilation during PDT (42.3 %, n = 119) insertion. Almost 80 % of respondents administered a combination of sedation, analgesia, and neuromuscular blockade to patients undergoing PDT insertion. Propofol was the most commonly used sedative overall (85.4 %, n = 240), with 92.3 % (n = 192) in E and 65.8 % (n = 48) OE. Fentanyl and remifentanil were the most used analgesic drugs overall, while fentanyl and morphine were the most used OE. Cis-atracurium and rocuronium were the neuromuscular blocking agents most used overall and in E, while atracurium and vecuronium were the most used OE. Local anesthesia was provided by 64.9 % (n = 179) of respondents. Bronchoscopy during PDT was used more frequently overall (69.2 %, n = 180); its use was more frequent in European ICUs than OE. Bronchoscopy during PDT was most often performed through the endotracheal tube (ETT) in place (86.9 %, n = 232), followed by the replacement of a larger ETT (6 %, n = 16) or laryngeal mask airway (LMA) 6 %, n = 16). The most used bronchoscope had a diameter of 5 mm overall (39.1 %, n = 86) and in E (42.9 %, n = 72) and 6 mm OE (38.5 %, n = 20). The bronchoscope was chosen more frequently according to the availability in the ICU (63.8 %, n = 143). Neck ultrasound was used more frequently in situations where other neck structures were considered to be at increased risk of injury (68.6 %, n = 81). A cuffed tube was the most frequently chosen tracheostomy tube at the end of the procedure (45.9 %, n = 118).

**Table 2:** Procedural features of percutaneous dilatational tracheostomy performed in the intensive care unit

	Response rate			Statistics
	Total	Europe	Outside Europe	
Who performed PDT in ICU				
- One or more intensivists	208/281 (74 %)	166/208 (79.8 %)	42/73 (57.5 %)	
- Intensivist with ENT specialists	16/281 (5.7 %)	10/208 (4.8 %)	6/73 (8.2 %)	
- Intensivist with general surgeon	6/281 (2.1 %)	1/208 (0.5 %)	5/73 (6.8 %)	*X* ^2^ = 51.732
- Intensivist with other surgeon	8/281 (2.8 %)	5/208 (2.4 %)	3/73 (4.1 %)	*P* = 0.000
- Anesthesiologist	18/281 (6.4 %)	17/208 8.2 %)	1/73 (1.4 %)	
- ENT specialists	15/281 (5.3 %)	7/208 (3.4 %)	8/73 (11 %)	
- General surgeon	7/281 (2.5 %)	1/208 (0.5 %)	6/73 (8.2 %)	
- Another surgeon	3/281 (1.1 %)	1/208 (0.5 %)	2/73 (2.7 %)	
Most frequent indication				
- Prolonged mechanical ventilation	151/281 (53.7 %)	107/208(51.4 %)	44/73 (60.3 %)	
- Difficult/prolonged weaning	68/281 (24.2 %)	55/208 (26.4 %)	13/73 (17.8 %)	*X* ^2^ = 9.039
- Neurocritical disease (medical, disease, surgical or trauma involving the neurologic system)	41/281 (14.6 %)	32/208 (15.4 %)	9/73 (12.3 %)	*P* = 0.171
- Inability to perform airway protection	12/281 (4.3 %)	8/208 (3.8 %)	4/73 (5.5 %)	
- Inability to cough and swallow	5/281 (1.8 %)	4/208 (1.9 %)	1/73 (1.4 %)	
- Improvement of patient respiratory mechanics	3/281 (1.1 %)	1/208 (0.5 %)	2/73 (2.7 %)	
- Copious secretions	1/281 (0.4 %)	1/208 (0.5 %)	0	
Most frequent timing				
- <7days	55/281 (19.6 %)	47/208 (22.1 %)	9/73 (12.3 %)	
- 7–15 days	153/281 (54.4 %)	108/208 (51.9 %)	45/73 (61.6 %)	*X* ^2^ = 9.707
- 15–21 days	58/281 (20.6 %)	43/208 (20.7 %)	15/73 (20.5 %)	*P* = 0.046
- 21–30 days	11/281 (3.9 %)	7/208 (3.4 %)	4/73 (5.5 %)	
- >30 days	4/281 (1.4 %)	4/208 (1.9 %)	0	
Mechanical ventilation mostly used				
- Volume control ventilation	119/281 (42.3 %)	84/208 (40.4 %)	35/73 (47.9 %)	
- Pressure control ventilation	102/281 (36.3)	75/208 (36.1 %)	27/73 (37 %)	
- Minute volume/adaptive support ventilation	13/281 (4.6 %)	12/208 (5.8 %)	1/73 (1.4 %)	*X* ^2^ = 15.504
- Bi-level airway pressure	34/281 (12.1 %)	30/208 (14.4 %)	4/73 (5.5 %)	*P* = 0.004
- Other	13/281 (4.6 %)	7/208 (3.4 %)	6/73 (8.2 %)	
Sedation-analgesia-neuromuscular blocking protocol provided				
- Yes	221/278 (79.5 %)	163/207 (78.7 %)	58/71 (81.7 %)	*X* ^2^ = 0.456
- No	57/278 (20.5 %)	44/207 (21.3 %)	13/71 (18.3 %)	*P* = 0.500
Local anesthesia provided				
- Yes	179/276 (64.9 %)	124/206 (60.2 %)	55/70 (78.6 %)	*X* ^2^ = 12.859
- No	97/276 (35.1 %)	82/206 (39.8 %)	15/70 (21.4 %)	*P* = 0.000
Bronchoscopy used during PDT				
- Yes	180/260 (69.2 %)	153/194 (78.9 %)	39/66 (59.1 %)	*X* ^2^ = 48.827
- No	80/260 (30.8 %)	41/194 (21.1 %)	27/66 (40.9 %)	*P* = 0.000
Bronchoscopy during PDT performed				
- Through ETT in place	232/267 (86.9 %)	176/201(87.6 %)	56/66 (84.8 %)	
- Replacement with larger ETT	16/267 (6 %)	12/201 (6 %)	4/66 (6.1 %)	*X* ^2^ = 0.807
- Replacement with smaller ETT	3/267 (1.1 %)	2/201 (1 %)	1/66 (1.5 %)	*P* = 0.848
- Through LMA	16/267 (6 %)	11/201 (5.5 %)	5/66 (7.6 %)	
- Diameter of bronchoscope used				
- 3–4 mm	40/220 (18.2 %)	34/169 (20.2 %)	6/52 (11.5 %)	
- 5 mm	86/220 (39.1 %)	72/169 (42.9 %)	14/52 (26.9 %)	*X* ^2^ = 17.268
- 6 mm	65/220 (29.5 %)	45/169 (26.8 %)	20/52 (38.5 %)	*P* = 0.001
- 7 mm	29/220 (13.2 %)	17/169 (10.1 %)	12/52 (23.1 %)	
- ≥8 mm	0	0	0	
Bronchoscope was chosen				
- According to availability in ICU	143/224 (63.8 %)	110/171 (64.3 %)	33/53 (62.3 %)	
- According the size of ETT in place	51/224 (22.8 %)	37/171 (21.6 %)	14/53 (26.4 %)	*X* ^2^ = 1.051
- Always with the smallest diameter	30/224 (13.4 %)	24/171 (14 %)	6/53 (11.3 %)	*P* = 0.591
- Randomly without assessment	0	0	0	
Neck ultrasound for PDT used				
- To guide needle, dilator and cannula placement	25/118 (21.2 %)	18/95 (18.9 %)	7/23 (30.4 %)	*X* ^2^ = 5.966
- In suspected at-risk structure	81/118 (68.6 %)	69/95 (72.6 %)	12/23 (52.2 %)	*P* = 0.051
- Both together	127118 (10.2 %)	8/95 (8.4 %)	4/23 (17.4 %)	
Tracheostomy tube used				
- Cuffed	118/257 (45.9 %)	89/194 (45.9 %)	29/63 (46 %)	
- Cuffed with inner cannula	94/257 (36.6 %)	75/194 (38.7 %)	19/63 (30.2 %)	*X* ^2^ = 6.568
- Both	43/257 (16.7 %)	28/194 (14.4 %)	15/63 (23.8 %)	*P* = 0.087
- Other	2/257 (0.8 %)	2/194 (1 %)	0	

*PDT* percutaneous dilatational tracheostomy, *ENT* ear-nose-throat, *ETT* endotracheal tube, *LMA* laryngeal mask airway

### Surgical tracheostomy

Table 3 describes the use of surgical tracheostomy (ST) in the ICU. STs were performed more frequently in the critically ill in ICU (59.1 %, n = 166), with only 15.6 % undergoing ST in the operation room (OR). In the OR, ST was performed more frequently by ear-nose-throat (ENT) specialists (59.7 %, n = 165) followed by general surgeons (14.9 %, n = 42). ST was more often reserved for patients who were deemed to be at higher risk of difficult PDT insertion (70.1 %, n = 187).

**Table 3:** Response rate for the subgroup of surgical tracheostomies

	Response rate			Statistics
	Total	Europe	Outside Europe	
Where ST was performed				
- ICU	106/281 (37.7 %)	74/208 (35.6 %)	32/73 (43.8 %)	*X* ^2^ = 3.220
- Operating room	166/281 (59.1 %)	128/208 (61.5 %)	38/73 (52.1 %)	*P* = 0.200
- Other	9/281 (3.2 %)	6/208 (2.9 %)	3/73 (4.1 %)	
Critically ill patients receiving Tracheostomy in operating room	2797 (15.6 %)*	2022 (15.2 %)	775 (16.2 %)	*P* = 0.642
Who performed ST in operating room				
- Anesthesiologist	2/281 (0.7 %)	2/208 (1 %)	0	
- Intensivist	7/281 (2.5 %)	6/208 (3 %)	1/73 (1.4 %)	
- ENT specialist	165/281 (58.7 %)	113/208 (56.8 %)	52/73 (73.2 %)	
- General surgeon	42/281 (14.9 %)	29/208 (14.6 %)	13/73 (18.3 %)	*X* ^2^ = 28.302
- Thoracic surgeon	17/281 (6 %)	15/208 (7.5 %)	2/73 (2.8 %)	*P* = 0.000
- Plastic surgeon	1/281 (0.4 %)	0	1/73 (1.4 %)	
- Maxillofacial surgeon	28/281 (10 %)	26/208 (13.1 %)	2/73 (2.8 %)	
- Other	8/281 (2.8 %)	8/208 (4 %)	0	
Why ST was chosen				
- Insufficient expertise in PDT	22/281 (7.8 %)	9/208 (4.3 %)	13/73 (17.8 %)	
- Reserved for patients with predicted difficult PDT	187/281 (70.1 %)	153/208 (73.6 %)	44/73 (60.3 %)	*X* ^2^ = 20.207
- Predicted need for prolonged tracheostomy	23/281 (8.2 %)	19/208 (9.1 %)	4/73 (5.5 %)	*P* = 0.000
- Other	39/281 (13.9 %)	27/208 (13 %)	12/73 (16.4 %)	

*Percentage calculated from total number of tracheostomies performed. *ST* surgical tracheostomy, *ENT* ear-nose-throat, *PDT* percutaneous dilatational tracheostomy

### Complications

The most frequent intra-procedural and early complications in E and OE was bleeding controlled by compression at 31.7 % (n = 89) followed by ETT puncture 20.2 % (n = 52). Bleeding requiring exploration was reported to be the most frequent late complication (33.1 %, n = 85). Table 4 shows the intra-procedural, early and late complications.

**Table 4:** Intraprocedural, early and late complications

	Response rate			Statistics
	Total	Europe	Outside Europe	
Most frequent intraprocedural complication				
- Puncture posterior tracheal wall	2/257 (0.8 %)	2/194 (1 %)	0	
- Puncture ETT	52/257 (20.2 %)	47/194 (24.2 %)	5/63 (7.9 %)	
- Accidental extubation	17/257 (6.6 %)	15/194 (7.7 %)	2/63 (3.2 %)	
- Difficult cannula placement	47/257 (18.3 %)	34/194 (17.5 %)	13/63 (20.6 %)	
- Stoma not adequate	6/257 (2.3 %)	1/194 (0.5 %)	5/63 (7.9 %)	*X* ^2^ = 36.296
- False passage	4/257 (1.6 %)	2/194 (1 %)	2/63 (3.2 %)	*P* = 0.000
- Convert the procedure	4/257 (1.6 %)	2/194 (1 %)	2/63 (3.2 %)	
- Bleeding controlled by compression	89/257 (31.7 %)	63/194 (32.5 %)	26/63 (41.3 %)	
- Bleeding requiring exploration	1/257 (0.4 %)	1/194 (0.5 %)	0	
- Desaturation (<90 %)	16/257 (6.2 %)	11/194 (5.7 %)	5/63 (7.9 %)	
- Pneumothorax	0	0	0	
- Emphysema	1/257 (0.4 %)	1/194 (0.5 %)	0	
- Other	18/257 (7 %)	15/194 (7.7 %)	3/63 (4.8 %)	
Most frequent early complication				
- Puncture posterior tracheal wall	1/257 (0.4 %)	1/194 (0.5 %)	0	
- Puncture ETT	16/257 (6.2 %)	14/194 (7.2 %)	2/63 (3.2 %)	
- Accidental extubation	3/257 (1.2 %)	3/194 (1.5 %)	0	
- Difficult cannula placement	13/257 (5.1 %)	10/194 (5.2 %)	3/63 (4.8 %)	
- Stoma not adequate	2/257 (0.8 %)	0	2/63 (3.2 %)	*X* ^2^ = 37.981
- False passage	2/257 (0.8 %)	2/194 (1 %)	0	*P* = 0.000
- Convert the procedure	2/257 (0.8 %)	2/194 (1 %)	0	
- Bleeding controlled by compression	163/257 (63.4 %)	114/194 (58.8 %)	49/63 (77.8 %)	
- Bleeding requiring exploration	9/257 (3.5 %)	9/194 (4.6 %)	0	
- Desaturation (<90 %)	11/257 (4.3 %)	10/194 (5.2 %)	1/63 (1.6 %)	
- Pneumothorax	1/257 (0.4 %)	1/194 (0.5 %)	0	
- Emphysema	8/257 (3.1 %)	4/194 (2.1 %)	4/63 (6.3 %)	
- Other	26/257 (10.1 %)	24/194 (12.4 %)	2/63 (3.2 %)	
Most frequent late complication				
- Bleeding controlled by compression	44/257 (17.1 %)	35/194 (18 %)	9/63 (14.3 %)	
- Bleeding requiring exploration	15/257 (5.8 %)	13/194 (6.7 %)	2/63 (3.2 %)	*X* ^2^ = 3.555
- Stoma infection/inflammation	85/257 (33.1 %)	61/194 (31.4)	24/63 (38.1 %)	*P* = 0.470
- Cannula extraction/malpositioning	54/257 (21 %)	40/194 (20.6 %)	14/63 (22.2 %)	
- Other	59/257 (23 %)	45/194 (23.2 %)	14/63 (22.2 %)	

## Discussion

This is the first international survey investigating the current spectrum of clinical practice in tracheostomy insertion in critically ill patients. In this investigation we found that single-step dilatational tracheostomy was the most frequent PDT approach used in the ICU. Intensive care physicians mainly performed PDT for prolonged mechanical ventilation. Consistent with this, the most popular timing for tracheostomy was between 7 and 15 days after ICU admission. Volume control mechanical ventilation, sedation-analgesia-neuromuscular blocking agents, local anesthesia and fiber optic bronchoscope were most frequently used during the procedure. ST was also frequently performed in critically ill patients. In this survey ST was mainly performed in the ICU by ENT specialists and was often chosen in cases where PDT was predicted to be difficult. In regard to complication arising from tracheostomy, our findings suggest that bleeding controlled by compression and stoma infection/inflammation were the most common intra-procedural and late complications, respectively. There was considerable variability in regard to practices surrounding informed consent for tracheostomy in critically ill patients, with informed consent for PDT obtained in only 60 % of cases.

This is the first survey investigating the international clinical practice for PDT in critically ill patients, because previous surveys have been national investigations performed in European countries [5–12]. In addition, none of the previous surveys reported information about PDT performed outside Europe. In this survey 26 % of respondents came from outside Europe. This allows us to evaluate the differences between tracheostomy performed in and outside Europe. Outside Europe: 1) the most used tracheostomy was the ST performed in the ICU with more involvement of ENT specialists; 2) informed consent for tracheostomy was obtained more than in Europe; 3) the intensivists more frequently performed one PDT, compared to at least two or more in Europe; 4) PDT was performed less in the first 7 days of admission; 5) a fiber optic bronchoscope was used less often, and a diameter >6 mm was used; 5) ST was chosen for patients with predicted difficulties for PDT but also where there was insufficient expertise in performing PDT; and 6) ETT puncture occurred less frequently as intra-procedural complications and there was increased incidence of subcutaneous emphysema among early complications.

In line with previous surveys, SSDT was the most popular technique in Europe and was mostly performed by intensivists [5–7]. Surprisingly, ST was the most popular tracheotomy technique outside Europe, and was mainly performed by ENT specialists. PDT and ST were easily performed, with a faster learning curve [5]. PDT is a safe alternative to ST [13]. However, ENT specialists and general surgeons preferred performing ST in the OR compared to PDT at the bedside [14]. The percutaneous approach was also a skill in the training of intensive care physicians, whereas surgeons largely performed open tracheostomies [15]. We think that the preference, background and training of physicians were responsible for the choice of the tracheostomy technique in critically ill patients. Indeed in this survey, ST was chosen more frequently outside Europe because of the insufficient expertise in performing PDT.

In our survey, prolonged mechanical ventilation followed by difficult weaning were frequent indications for PDT. This result was in line with previous surveys and reports in the literature. Tracheostomy in the ICU was typically indicated when patients were unlikely to undergo successful extubation [16]. In this case, the risk of tracheostomy outweighed the risk of prolonged translaryngeal intubation [16]. The most frequent timing of PDT insertion in this international survey was reported as 7–15 days; this is in line with previous national surveys [5–12]. Recent trials did not report any improvement in mortality after 30 days comparing early (<4 days) and late (>10 days) tracheostomy [17] or onset of pneumonia with the cutoff between early and late tracheostomy at 13 days. In our opinion the timing of PDT should be individualized according to patients’ needs.

Volume control ventilation was the most used in and outside Europe. We found increased use of manual ventilation outside Europe during the procedure. Sedation, analgesia and neuromuscular blocking protocols were mostly used in and outside Europe in line with the previous Italian survey [5]. Interestingly, local anesthesia was more frequently provided outside Europe, probably due to the high incidence of ST. However, the use of general anesthesia with local anesthesia or of local anesthesia alone differed in and outside Europe according to the type of physician performing the tracheostomy. Bronchoscopy during PDT was used more in Europe than outside Europe. The frequent use of bronchoscopy in Europe should be linked to more PDT being performed. PDT is a blind technique for obtaining a surgical airway [18], therefore the use of bronchoscopy may add more safety to this procedure [19]. Bronchoscopy outside Europe was used by 59 % of respondents, probably due to ST being used more frequently. ST is an open procedure with direct visualization of procedural steps [20]. In this case use of the bronchoscope may be avoided. In Europe the respondents chose a bronchoscope with small diameters compared with those chosen outside Europe. Minimizing the diameter of the bronchoscope during PDT may reduce airway obstruction and hypercarbia or hypoxia [21, 22]. However, a new tool for PDT has been recently proposed [23], which allows bronchoscopy without compromising ventilation. In recent years, the role of bronchoscopy in PDT has been questioned and the use of neck ultrasound has been suggested [18]. However, in this survey the neck ultrasound was used more frequently in situations where other neck structures were considered to be at increased risk of injury.

Bleeding controlled by compression and ETT puncture were the most common intra-procedural and early complications found in Europe, while there was a lower incidence of ETT puncture outside Europe. Bleeding and airway complications were the main complications related to PDT [24]. Outside Europe respondents reported lower ETT puncture rates, likely because of the higher incidence of open ST, allowing direct visualization of the overall procedure.

Informed consent for PDT was obtained in 60 % of cases and it was obtained more frequently outside Europe. Indeed, informed consent before invasive and surgical procedures has become standard practice in many medical institutions [25]. In a national survey on informed consent for tracheostomy in Italy we found that informed consent for this procedure was provided by 65 % of patients undergoing tracheostomy [26]. The practice of obtaining informed consent varied in different countries according to national legislation, history, culture, and religion [27]. Tracheostomy is an elective procedure committing the patients to a prolonged period of recovery from critical illness. The choice of performing tracheostomy in critically ill patients should be carefully discussed by ICU physicians and then with patients or the patients’ families.

There appears to be considerable variability in tracheostomy practices in critically ill patients. For European tracheostomy, our survey documented a general agreement with respect to the indications, complications and procedural steps for PDT and ST. The common methodology of practice is similar to published protocols, such as the recent national Danish guideline for percutaneous tracheostomy in the ICU [28]. Outside of Europe, such respondents constituted approximately one third of participants and their responses were heterogeneous and frequently country-specific, illustrating that there are different styles of practice in international clinical practice. The results of our survey may be an initial step towards promoting a unified international practice guideline for tracheostomy in the ICU.

### Limitations

This study suffers from some potential limitations. We found many differences between respondents in Europe and outside Europe in the baseline characteristics of the type of institution, type of intensive care, number of ICU beds and different techniques performed. We also performed statistical analysis according to the type of intensive care, type of institution and different techniques performed, but the results did not change. Respondents from outside Europe represented only one third of participants. For this reason we have added weighting for the total number of respondents in Europe and outside Europe to perform tests of statistical significance. The respondents outside Europe were heterogeneous, varying between different countries. This survey was the first to look at the international practice of tracheostomy. We were unable to further investigate other aspects of clinical practice, such as the use of ultrasound and/or LMA, in order to avoid too lengthy a survey.

## Conclusions

PDT is used worldwide in critically ill patients, without clinical guidelines to suggest the best practice [3]. This survey represents the first snapshot of PDT international clinical practice. This first global picture of current practices in regard to tracheostomy insertion demonstrates considerable international variation in practice, suggesting a need for greater standardization of approaches to tracheostomy.

## Key messages

- Percutaneous dilatational tracheostomy is one of the most frequent procedure performed in the intensive care unit
- Percutaneous dilatational tracheostomy is used worldwide in critically ill patients, without clinical guidelines to suggest the best practice
- This survey represents the first global picture of current practices in performing tracheostomy
- This survey demonstrates considerable international variation in practice, suggesting a need for greater standardization of approaches to tracheostomy insertion

## Additional files

Additional file 1:
**Questionnaire proposed to European Society of Intensive Care Medicine (ESICM) members.** (PDF 79 kb) Additional file 2:
**Response rate divided according to geographical localization.** (PDF 71 kb)

## Abbreviations

E: Europe
ENT: ear-nose-throat
ESICM: European Society of Intensive Care Medicine
ETT: endotracheal tube
LMA: laryngeal mask airway
OE: outside Europe
OR: operation room
PDT: percutaneous dilatational tracheostomy
SSDT: single-step dilation tracheostomy
ST: surgical tracheostomy
