# Supplementary material for: Tracheostomy procedures in the intensive care unit: an international survey
Source: Crit Care. 2015 Aug 13;19(1):291. doi: 10.1186/s13054-015-1013-7 (PMC4536803; doi:10.1186/s13054-015-1013-7)
Supplement: Additional file 1: — Questionnaire proposed to European Society of Intensive Care Medicine (ESICM) members. (PDF 79 kb) [file 13054_2015_1013_MOESM1_ESM.pdf]

Questionnaire:
TRACHEOSTOMY PROCEDURES IN INTENSIVE CARE UNIT: A WORLDWIDE SURVEY

1) In which country do you practice? (Add your city and the name of your Intensive Care Unit)

2) What is your main specialty area?
- Intensive Care
-Anesthesiology
-Pulmonology
-Cardiology
-Neurology
-Surgery
- Other

3) What type of Institution is your hospital:
-Public hospital
-Private hospital
-University hospital

4) What is the type of Intensive Care that you work in (tick all that apply)?
-Neurological ICU
-Medical ICU
-Surgical ICU
-Cardiac ICU
-Mixed ICU

5) What is the number of beds in your ICU:
- ≤ 5
- 6-10
- 11-15
- 16-20
- ≥21

6) What is the approximate number of patients/year admitted to your ICU:
- ≤300
- 301- 600
- 601- 999
- ≥1000

7) What is the number of tracheostomies that were performed in your ICU in 2012?

8) What number tracheostomies were performed in your ICU in the 2012 using the following techniques:
- Ciaglia single dilator
- Ciaglia multiple dilator
- Guide wire dilating forceps – Griggs
- Rotational dilation technique (PercuTwist)
- Balloon dilation technique (Ciaglia Blue-dolphin)
- Translaryngeal tracheostomy
- Surgical tracheostomy
- Other (specify)

9) Do you obtain an informed written consent for tracheostomy?
- Yes
- No

10) Where are surgical tracheostomies s usually performed:
- ICU
- Operation room
- Other (Specify)

11) How many ICU patients had their tracheostomies performed in the operating room in the 2012?

12) Who most commonly performs the tracheostomy procedure in ICU?
- One or more ICU physicians 1
- Intensivists with ENT specialist assistance 2
- Intensivists with the assistance of a general surgeon 3
- Intensivists with the assistance of other surgical specialists 4
- Anesthesiologists 5
- An ENT specialist 6
- A General surgeon 7
- Another surgeon (specify) 8

13) Who performs the surgical tracheostomy in operation room?
- Anesthesiologists 1
- ENT specialists 2
- Intensivists 3
- General surgeon 4
- Thoracic surgeon 5
- Plastic surgeon 6
- Maxillofacial surgeon 7
- Trauma surgeon 8
- Other (specify) 9

14) Why do you choose surgical tracheostomy?
- Insufficient expertise in Percutaneous Tracheostomy
- Surgical approach reserved for patients with predicted difficult percutaneous tracheostomy
- Surgical approach reserved for patients with predicted need for prolonged tracheostomy

Other reason (please specify):

15) The most frequent indication for tracheostomy in your ICU is: (single choice)
- Prolonged mechanical ventilation[*]
- Difficult/prolonged weaning[+]
- Neurocritical disease (medical, surgical or trauma)
- Inability to airway protection
- Inability to cough and swallow
- Improvement of patient respiratory mechanics
- Copious secretions

[*] Prolonged mechanical ventilation has been defined as a period of 21 days or more.

[+] Difficult/prolonged weaning has been defined as weaning requiring ≥ 3 spontaneous breathing trials or failure of ≥ 3 weaning attempts or requiring > 7 days after the first attempt.

16) The most frequent timing for tracheostomy in your ICU is:
(timing is referred to days post tracheal intubation)
- <7 days
- 7-15 days
- 15-21 days

-21-30 days
- >30 days

17) Mechanical ventilation mostly used during tracheostomy in your ICU:
- Volume controlled ventilation
- Pressure controlled ventilation
- Minute volume ventilation/ Adaptive support ventilation
- Bi-level airway pressure
- Other (specify)

18) A sedation-analgesia-neuromuscular blocking protocol is provided for tracheostomy in your ICU?
- Yes
- No

19) Drugs used for sedation during percutaneous tracheostomy procedures: (multiple choices)
- Diazepam
- Lorazepam

- Midazolam
- Propofol
- Other sedative medication (specify)

20) Drugs used for analgesia during percutaneous tracheostomy procedures:
- Alfentanil
- Fentanyl
- Morphine
- Remifentani
- Sufentanil
- Other analgesic medication (specify)

21) Drugs used for neuromuscular blockade during percutaneous tracheostomy procedures:
- Atracurium
- Cis-atracurium
- Rocuronium
- Succinylcholine
- Vecuronium
- Other neuromuscular blocking (specify)

22) Is local anesthesia provided for percutaneous tracheostomy procedures?
- Yes
- No

23) Is fiber-optic bronchoscope used during percutaneous tracheostomy procedures?
- Yes
- No

24) Bronchoscopy during percutaneous tracheostomy is performed:
- through the ETT in place in the patient
- through a replacement that is larger ETT than the one in place in the patient
- through a replacement that is smaller ETT than the one in place in the patient
- through a laryngeal mask airway

25) The diameter of the fiber-optic bronchoscope used during percutaneous tracheostomy is:
- 3-4 mm
- 5 mm
- 6 mm
- 7 mm
- ≥ 8 mm

26) The fiber-optic bronchoscope that is used is:

- chosen according the availability of bronchoscopes in the ICU
- chosen according the size of ETT in place in the patient
- In all the cases the bronchoscope with the smallest diameter is chosen
- The Bronchoscope is chosen randomly without any assessment

27) Is ultrasound (US) evaluation of neck tissue performed for tracheostomy:
- We use neck US in all procedures in order to guide needles, dilators, and cannula
- We use US only if we suspect the presence of at-risk structure
- In case of at-risk structure, we use neck ultrasound to guide needles, dilators and cannula

28) Which tracheostomy tube do you use?
- Cuffed tube
- Cuffed tube with inner cannula
- Both
- Other (Specify)

29) The most frequent complication during the tracheostomy procedure in your ICU is:
- Puncture of posterior tracheal wall
- Puncture of tracheal tube
- Accidental extubation during the procedure
- Difficult placement of the cannula
- Tracheal stoma not adequate
- False passage of seldinger and cannula
- Necessity to convert a procedure into another
- Bleeding controlled by compression
- Bleeding requiring exploration
- Desaturation (SaO_2 < 90%)
- Pneumothorax
- Subcutaneous emphysema
- Other (Specify)

30) The most frequent early (in the first 24 hours) complication of the tracheostomy procedure in your ICU is:
- Puncture of posterior tracheal wall
- Puncture of tracheal tube
- Accidental extubation during the procedure
- Difficult placement of the cannula
- Tracheal stoma not adequate
- False passage of seldinger and cannula
- Necessity to convert a procedure into another
- Bleeding controlled by compression

- Bleeding requiring exploration
- Desaturation (SaO2 < 90%)
- Pneumothorax
- Subcutaneous emphysema
- Other (Specify)

31) The most frequent late (from the 2nd day to the discharge from the ICU) complication of the tracheostomy procedure in your ICU is:
- Bleeding controlled by compression
- Bleeding requiring exploration
- Stoma infections/inflammations*
- Cannula extraction/malpositioning
- Other (Specify)

* Stoma infections/inflammations has been defined as sign of inflammation and purulent discharge of the stoma
