# Supplementary figures and images for: Tracheostomy procedures in the intensive care unit: an international survey
Source: Crit Care. 2015 Aug 13;19(1):291. doi: 10.1186/s13054-015-1013-7 (PMC4536803; doi:10.1186/s13054-015-1013-7)

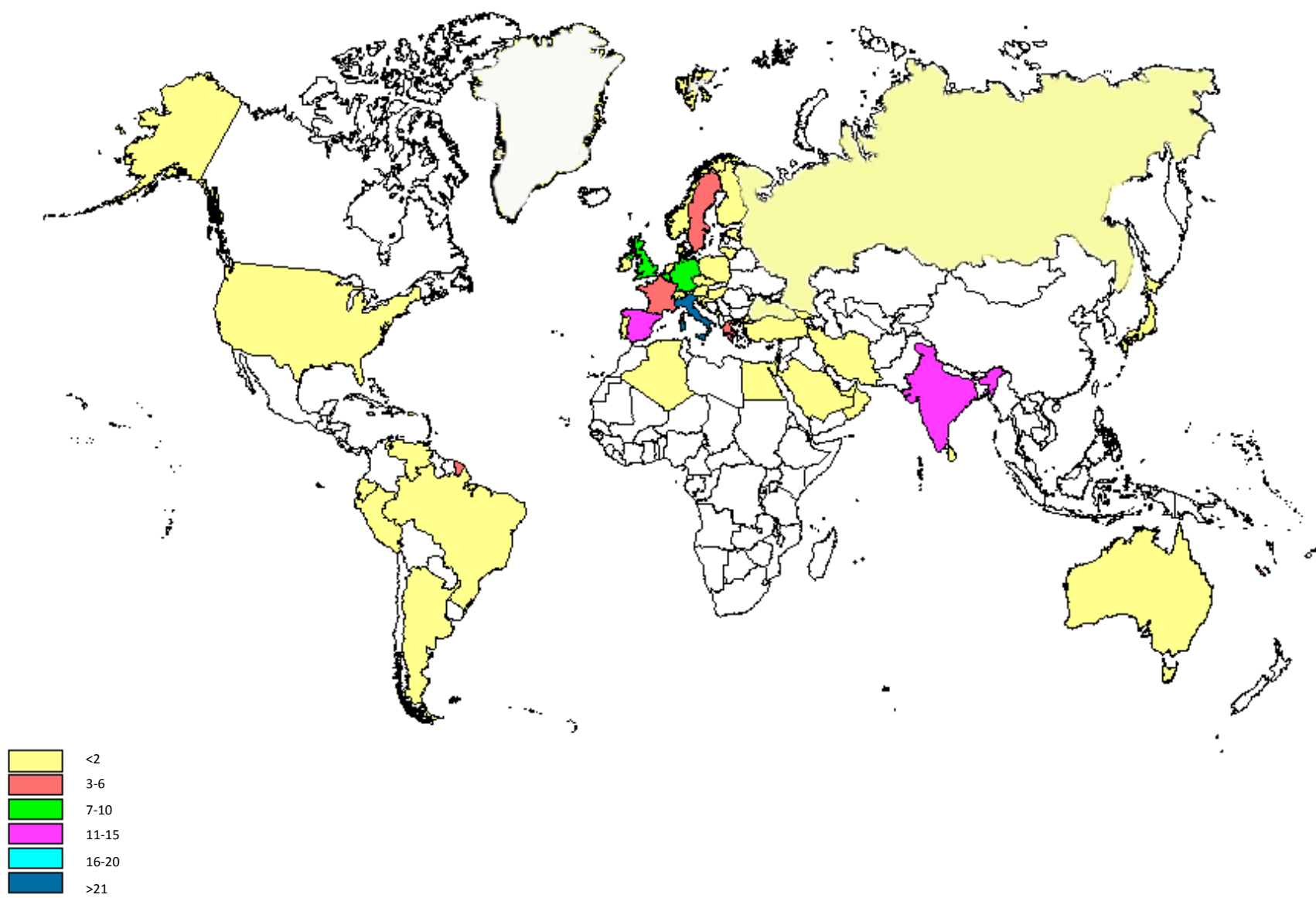

Supplement: Additional file 2: — Response rate divided according to geographical localization. (PDF 71 kb) [file 13054_2015_1013_MOESM2_ESM.pdf]
